# The Bee Hemolymph Metabolome: A Window into the Impact of Viruses on Bumble Bees

**DOI:** 10.3390/v13040600

**Published:** 2021-04-01

**Authors:** Luoluo Wang, Lieven Van Meulebroek, Lynn Vanhaecke, Guy Smagghe, Ivan Meeus

**Affiliations:** 1 Guangdong Provincial Key Laboratory of Insect Developmental Biology and Applied Technology, Institute of Insect Science and Technology, School of Life Sciences, South China Normal University, Guangzhou 510610, China; Luoluo.Wang@scnu.edu.cn; 2 Department of Plants and Crops, Faculty of Bioscience Engineering, Ghent University, 9000 Ghent, Belgium; Guy.Smagghe@UGent.be; 3 Laboratory of Chemical Analysis, Department of Veterinary Public Health and Food Safety, Faculty of Veterinary Medicine, Ghent University, 9820 Merelbeke, Belgium; Lieven.Vanmeulenbroek@UGent.be (L.V.M.); Lynn.Vanhaecke@UGent.be (L.V.)

**Keywords:** metabolomics, biomarker, Israeli acute paralysis virus, slow bee paralysis virus, bombus terrestris

## Abstract

State-of-the-art virus detection technology has advanced a lot, yet technology to evaluate the impacts of viruses on bee physiology and health is basically lacking. However, such technology is sorely needed to understand how multi-host viruses can impact the composition of the bee community. Here, we evaluated the potential of hemolymph metabolites as biomarkers to identify the viral infection status in bees. A metabolomics strategy based on ultra-high-performance liquid chromatography coupled to high-resolution mass spectrometry was implemented. First, we constructed a predictive model for standardized bumble bees, in which non-infected bees were metabolically differentiated from an overt Israeli acute paralysis virus (IAPV) infection (R^2^Y = 0.993; Q^2^ = 0.906), as well as a covert slow bee paralysis virus (SBPV) infection (R^2^Y = 0.999; Q^2^ = 0.875). Second, two sets of potential biomarkers were identified, being descriptors for the metabolomic changes in the bee’s hemolymph following viral infection. Third, the biomarker sets were evaluated in a new dataset only containing wild bees and successfully discriminated virus infection versus non-virus infection with an AUC of 0.985. We concluded that screening hemolymph metabolite markers can underpin physiological changes linked to virus infection dynamics, opening promising avenues to identify, monitor, and predict the effects of virus infection in a bee community within a specific environment.

## 1. Introduction

Viruses are etiological agents of diseases and also infect bee species [1]. Understanding the health impacts of virus–bee interactions, to ultimately identify dangerous bee viral diseases is critical to safeguard bees [2,3]. The current diagnostic arsenal of virus infection in bees exhibits high sensitivity (e.g., high-throughput sequencing can detect down to a single targeted molecule), and new viral sequences are being reported at a higher rate than ever before [4,5,6]. However, this raises two questions about bee viral disease monitoring. First, current methods are predominantly based on pathogen-detection (e.g., binding properties of antisera and the nucleic acid-based sequencing) [1,6,7,8,9], providing limited insights into the diagnosis of viral disease. In fact, in any bee population or individual under investigation, viruses are infectious [10,11]. Hence, the detection of viral presence is not equal to viral disease. Second, there is growing evidence that covert virus infections in bees is more common than previously expected [1,12], whereby a covert infection can turn into an overt infection under specific immune-suppressed conditions [13,14]. For instance, the covert infection by deformed wing virus (DWV) represents a sword of Damocles permanently threatening the survival of honey bee colonies, and any factors affecting the honey bee’s antiviral defences can turn this pathogen into a dangerous killer [13]. Having techniques that can identify/describe these tipping points will allow us to better understand viral disease progression. To summarize, we need more advanced technologies to measure the bees’ health or physiological status, to catch up with, and to supplement the current state of the art viral detection techniques.

Different “omics” technologies exist that describe a specimen’s condition by generating a massive amount of data. Big data has been suggested to develop predictive models, for instance, to develop a diagnostic algorithm to identify specimens suffering from some disease [15]. The metabolome has become widely accepted as a dynamic and sensitive measure of the phenotype of a species at the molecular level [16,17], and can complement viral discovery screenings as it provides also insight into the bee’s physiological status. In addition, viruses are known to influence the metabolism at the cellular level, thereby disturbing homeostasis of the host metabolism [18,19]. Moreover, for the bumble bee *Bombus terrestris*, a metabolic biomarker-based approach showed significant potential to predict artificial food stress in worker bees [20]. With this rationale, the objective of this study was to evaluate whether the viral infection status of the bee can be defined based on the metabolic fingerprint of the bee’s hemolymph.

We used standardized commercial *B. terrestris* and performed a biomarker discovery study for the Israeli acute paralysis virus (IAPV) and the slow bee paralysis virus (SBPV). Injection of IAPV in bumble bees leads to high bee mortality within the first week after injection, while SBPV-infected bees survive, although viral titers increase [21]. An untargeted metabolomics approach was implemented, based on generic extraction and ultra-high-performance liquid-chromatography coupled to high-resolution mass spectrometry, resulting in a multivariable dataset of unidentified metabolites. We specifically searched for those metabolites that were most robust to predict a viral infection status. This was achieved by introducing an additional stressor (i.e., malnutrition) and evaluating its influence on the predictive power of the model. In addition, we also implemented a targeted approach that allowed a more mechanistic evaluation of metabolic shifts in the bee hemolymph in response to the virus, based on metabolite identities. Establishing a predictive model for standardized bees is considered only a first step towards monitoring viral damage in bumble bees in natural environments as one can presume that biomarkers lose their predictive value as the number of variables and additional stressors increase. Therefore, the predictive value of different biomarker sets was tested for a population containing only wild bees. We speculate that biologically verified biomarkers, if supported by literature [19,22,23], will be more likely to maintain their predictive power in wild bumble bees or to provide some fundamental insights into why predictive power is lost.

## 2. Materials and Methods

### 2.1. Insect, Virus, and Injection 

All bees had a random age. Commercial bumblebees were randomly picked from indoor mass-reared queen-right colonies of *B. terrestris* (Biobest, Westerlo, Belgium). Wild B. terrestris worker bees were caught in urban areas of Ghent, Belgium during June 2019. Upon arrival in the lab, five random worker bees (commercial or wild) were placed in one microcolony. In total, we started 48 microcolonies (40 with commercial and 8 with wild-caught bees). These microcolonies were placed in an incubator at 30 °C, 60% relative humidity, and continuous darkness, and were all fed with gamma-irradiated pollen (Apihurdes, Pinofranqueado, Spain); 20 + 4 microcolonies (commercial or wild) received a standardized sugar syrup (50 *w/v* %, BIOGLUC, Biobest), while the other 20 + 4 microcolonies (commercial or wild) received a 25% sugar syrup to mimic a low carbohydrate nutritional stress [24]. Bees were kept in these conditions for one week. To establish a systematic infection, the virus was injected into the bee’s hemocoel using a Femtojet Microinjector (Eppendorf, Hamburg, Germany). IAPV and SBPV inocula were produced by propagating virus reference isolates in 50 white-eyed honeybee pupae and preparing a chloroform-clarified extract in phosphate buffer saline (PBS) (10 mM phosphate buffer (pH 7.0)/0.02% diethyl dithiocarbamate) by following the same protocol as described previously [25]. Prior to injection, the IAPV stock was diluted 10,000 times and the SBPV stock was diluted 50 times in a filter-sterilized phosphate buffer saline (PBS). Bees were firstly immobilized on ice for 2 min, and 5 µL of IAPV (~500 virus particles), 5 µL of SBPV (~1 × 10^5^ virus particles), or 5 µL of filter-sterilized PBS was injected through the membrane between the second and third segment in the abdomen. The IAPV and SBPV stocks were provided by Joachim de Miranda (Swedish University of Agricultural Sciences, Uppsala, Sweden). Both virus stocks were stored in aliquots at –80 °C and had an estimated number of 1 × 10^6^ particles per mL (measured by transmission electron microscope). In addition, virus stocks had < 0.1% contamination of other common honeybee viruses, as determined by RT-qPCR [26]. 

### 2.2. Hemolymph Collection 

Bee hemolymph was collected by making a small incision in the dorsal thorax and extracting 10 μL per bee using Wiretrol II Capillary micropipettes (VWR) into an Eppendorf tube that contained 2 μL of 5% N-phenylthiourea (PTU, *w/v*; Sigma Aldrich, Overijse, Belgium) in 1 mL of 50% methanol/PBS to prevent melanization. The hemolymph sample was collected on ice and immediately put on dry ice afterwards. All hemolymph collection was performed under binocular microscope, and three rules were strictly followed to guarantee the quality of sampling: (i) the hemolymph should be pure and transparent; (ii) no other tissues were perforated; (iii) sampling time (incision and extraction) per bee is less than 35 s. All samples were stored at −80 °C until chemical analysis. 

### 2.3. Extraction of Polar and Medium Polar Metabolites from Bee Hemolymph

Polar and medium polar metabolites were extracted from bee hemolymph as previously described [20]. In brief, 40 μL of methanol-ethyl acetate mixture was used for extraction of polar metabolites and precipitation of proteins. In addition, 5 μL valine-d8 internal standard solution (ISTD, 25 ng/μL in ultrapure water) was pre-added. Subsequently, samples were incubated for 30 min at 4 °C to enhance protein precipitation and centrifugated at 15,000× *g* for 15 min at 4 °C to remove the resulting precipitate. Ultimately, the supernatant was transferred to a 1.5-mL microfuge tube and consecutively dried using the Speed-Vac. All dried samples were suspended in 100 μL ultrapure water and transferred to an liquid-chromatography–mass spectrometry (LC-MS) vial with glass insert. Solvents used for extraction of hemolymph metabolites were of LC-MS grade, obtained from Fisher Scientific (Loughborough, UK) and VWR International (Merck, Darmstadt, Germany). Ultrapure water was obtained by usage of a purified-water system (VWR International, Merck).

### 2.4. UHPLC-Q-Orbitrap-HRMS Analysis

For ultra-high-performance LC hyphenated to Quadrupole-Orbitrap high-resolution MS (UHPLC-Q-Orbitrap-HRMS) analysis, the methodology used was as previously described [18]. The chromatographic separation was performed using an Ultimate 3000 XRS UHPLC system (Thermo Fisher Scientific, San José, CA, USA). An Acquity UPLC HSS-T3 column (1.8 μm, 150 mm × 2.1 mm) (Waters, Manchester, UK), kept at 45 °C, was used, to which a binary solvent system consisting of ultrapure water (A) and acetonitrile (B), with both acidified with 0.1% formic acid, was applied at a constant flow rate of 0.4 mL/min. All solvents used were of LC-MS grade and obtained from Fisher Scientific and VWR International (Merck). Ultrapure water was obtained by usage of a purified-water system (VWR International, Merck). Mass analysis was performed on a Q-ExactiveTM Orbitrap mass analyzer (Thermo Fisher Scientific) that was equipped with a heated electrospray ionization (HESI II) source, operating in polarity switching mode. Hereby, full-scan events were applied at a mass resolution of 70,000 full width at half maximum. A pool of all extracts (*n* = 150) was used to make quality control (QC) samples for instrument conditioning and data normalization. Experimental samples were run in a randomized order, except for QC samples, which were analyzed in duplicate after every nine experimental samples.

### 2.5. Predictive Modelling of Viral Infection in Standardized Bees

#### 2.5.1. Experimental Setup

We randomly assigned 200 commercial bees to 40 microcolonies consisting of four treatment groups, including PBS-injected bees, IAPV-injected bees, SBPV-injected bees, and mock bees (without any treatment). For each group, we also introduced the stressor malnutrition (bees fed on either normal 50% or stress 25% sugar syrup). In total, we sampled 110 bees for hemolymph extraction (i.e., in total three bees per microcolony one at day 2, day 4, and day 14 post virus injection, except for IAPV treated bees where we had only two sampling points since bees are dead 7 days post injection).

The choice of our sampling timepoint was based on previously established infection dynamics for IAPV and SBPV in commercial bumble bees. IAPV has a quick replication, resulting in high mortality in the first week, while SBPV has a steep increase in the first week, with a more moderate growth later and no mortality [21]. We opted to have a sampling point in an early and a later stage of the infection. We sampled IAPV from early infected bees 2 days post infection (2DPI, no dead bees at that moment) and late infected bees 4 days post infection (4DPI, with a fraction of bees already dead), and only considering bees without clear paralysis symptoms. For SBPV-infected bees, we selected the same sampling times of 2DPI and 4 DPI as these can be considered as the early stages of the infection. In addition, for SBPV we also sampled bees at 14 days post-infection (14DPI) as the late stage of infection. 

#### 2.5.2. Data Analysis and Biomarker Selection

Untargeted data analysis was performed as previously described by Wang et al. 2019 [19]. Briefly, LC-MS raw data were imported into Sieve 2.1 software package (Thermo Fisher Scientific) for peak extraction and alignment, deconvolution, and noise removal. This rendered a list of metabolite components (clustered features). The coefficient of variation (CV) was calculated for each component in the collection of QC samples, and only components with CV lower than 30%, which is considered an acceptable value of repeatability in untargeted metabolomics, were retained [27,28]. To correct for potential instrumental drift during analysis, the abundance of each component in a sample was divided by the corresponding mean abundance, as calculated for the two QC samples following that sample [29]. To assess the metabolic differences between the samples sets, unsupervised principal component analysis (PCA-X) and supervised pairwise OPLS-DA analyses were performed using SIMCA^®^ 14.1 multivariate statistics software (Umetrics, Malmo, Sweden). Model validation was performed through various quality parameters such as CV-ANOVA (*p*-value < 0.05), permutation testing (*n* = 100), Q^2^ (>0.5), and R^2^Y (>0.5) [30]. S-plots were built using validated OPLS-DA models to select components that are important for classifying bees with different levels of viral infection. Additional filtering of components was achieved using the variable importance in projection (VIP) score, for which a threshold of >1.5 was set. 

Targeted data analysis was performed using XcaliburTM 2.1 (Thermo Fisher Scientific), allowing quantitative analysis (peak integration) of metabolites that were identified based on the mass spectrum (*m*/*z*-value and 13C isotope profile) and retention time. Hereby, the identification process was based on an in-house library of analytical standards (±300 compounds), which were injected through various mixtures at the beginning of the analytical sequence. Statistical analysis was performed using SPSS 22.0 software, applying two-way ANOVA and Tukey HSD test for post-hoc comparisons, whereby a *p*-value < 0.05 was considered as statistically significant. Further filtering of candidate biomarkers was based on biological qualification, performing literature survey and metabolic pathway analysis (MetaboAnalyst, http://www.metaboanalyst.ca/ (accessed on 1 March 2021)). To judge the overall diagnostic efficacy of the selected metabolites in the standardized bee dataset, we calculated the AUC value using ROC curve analysis in MetaboAnalyst.

### 2.6. Predictive Potential of 2 Sets of Biomarkers in Wild Bumble Bees

The 40 wild caught *B. terrestris* were randomly assigned to the four treatment groups, including PBS-injected bees, IAPV-injected bees, SBPV-injected bees, and mock bees. To evaluate the predictive power of the candidate biomarker sets, we used different machines learning classification algorithms, performed in Waikato Environment for Knowledge Analysis (WEKA, version 3.8.4) [31]. The selected classifier was the random forest algorithm. Models were built with a single biomarker set or a combination of two biomarker sets. The 10-fold cross validation method was used during the training process, and then the classification accuracy was computed to represent the overall performance for each model. 

## 3. Results and Discussion 

**Figure 1:**
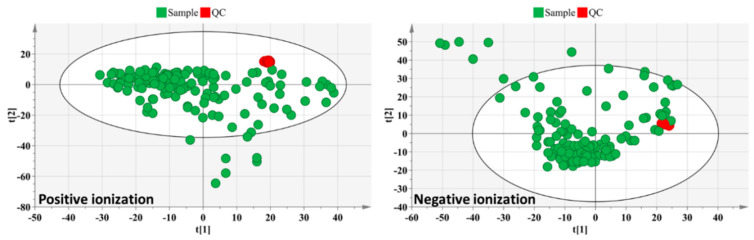
Principal component analysis (PCA-X) score plot of all analyzed samples (*n* = 150) of bee hemolymph after virus infection. PCA-X score plot is presented separately for positive ion mode (left) and negative ion mode (right). Red and green colors represent internal quality control (QC) and experimental samples, respectively. Associated PCA-X models were established based on non-normalized data.

Upon analysis of 150 hemolymph samples, obtained from B. terrestris hemolymph following different virus infections, nutritional stresses, and sample resources (commercially available and wild-caught), a total of 1149 (+ionization mode) and 911 (−ionization mode) components were defined as constituents of the metabolic fingerprint. The PCA-X score plots revealed good clustering of the internal QC samples (Figure 1), confirming good instrumental stability during sample analysis. 

### 3.1. Untargeted Data Analysis

In Table 1, we present an overview of OPLS-DA model performance in the commercial bumble bee dataset (*n* = 110). We performed multiple pairwise comparisons of various treatment groups.

#### 3.1.1. Predictive Modelling of Viral Infection in Non-Stressed Commercial Bees

We first analyzed if the viral infection status can be discriminated in standardized commercial bees, without any additional stressor, as stressors could potentially obscure clear virus related metabolome fingerprints. In bees receiving 50% sugar water, models had a predictive power to (1) find an overt (IAPV) and covert (SBPV) infection (Table 1, model 5 for IAPV, model 9 for SBPV) and (2) discriminate between the two viruses (Table 1, model 15). These results revealed that UHPLC-Orbitrap-MS-based metabolomics could successfully establish and distinguish the metabolic fingerprints of virus-infected bees from that of the non-virus-infected PBS controls, even in the asymptomatic SBPV covertly-infected bees.

#### 3.1.2. Predictive Modelling of Viral Infection in Stressed Commercial Bees

To select a robust set of biomarkers that have the potential to identify virus infection status in wild bees, the predictive framework must work in bees that are confronted with different stressors. To simulate this, we added a nutritional stressor to the standardized bee test group. We chose nutritional stress, which has been demonstrated to have synergistic effects to virus infection [33,34]. When bees were subjected to this diet stress, discrimination according to bee’s infection status was no longer possible (Table 1, model 4 for IAPV, model 8 for SBPV). This substantiates the results of our previous study; a 25% sugar concentration may cause significantly disturbed metabolic effects in the bee hemolymph [20]. Interestingly, when we combined the 25% and 50% sugar water treatment, the models regained their predictive power to (1) find a viral infection (Table 1, model 7 for IAPV, model 13 for SBPV); (2) discriminate between viruses (Table 1, model 7 for IAPV, model 16 for SBPV); and (3) discriminate between early and late infection stage (Table 1, model 6 for IAPV, model 10–12 for SBPV). 

#### 3.1.3. Biomarker Selection to Discriminate Infection in Wild Bees

**Figure 2:**
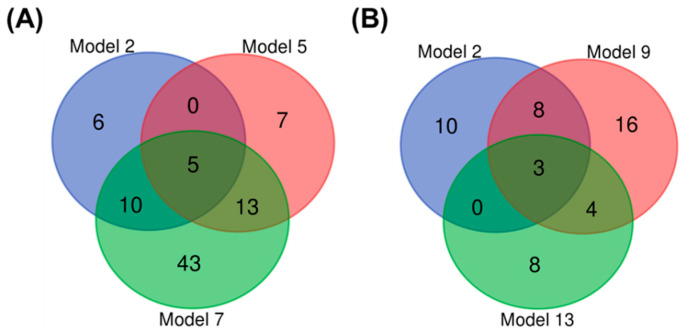
Biomarker Venn diagram. (**A**) Five Israeli acute paralysis virus (IAPV)-related biomarkers: the overlap between three circles defines the number of markers chosen by model 2 (virus vs. non-virus), model 5 (IAPV vs. phosphate buffer saline (PBS), with 50% diet), and model 7 (IAPV vs. PBS, all). (**B**) Three slow bee paralysis virus (SBPV)-related biomarkers: the overlap between three circles defines the number of markers chosen by model 2 (virus vs. non-virus), model 9 (SBPV vs. PBS, with 50% diet), and model 13 (SBPV vs. PBS, all). The general idea behind this selection is to select the most robust biomarkers related to IAPV or SBPV infection, even with variables from diets.

Although we cannot discriminate the viruses in stressed (malnourished) bees, our results confirmed that UHPLC-Orbitrap-MS-based metabolomics can clearly distinguish bees with or without virus infection in many situations (from single virus-infected bees to a mixture of single- and double-stressed bees). To select robust biomarkers, we defined components with discriminative power (meaning VIP > 1.5 in predictive models of Table 1). For each virus we selected those markers that were able to (1) identify viral infection (model 2), (2) identify which virus infected (models 5 and 9), and (3) identify which virus infected in the dataset including stressed bees (model 7 and 13). The predictive power (Q^2^) of model 13 is lower, meaning that including stressed bees indeed make the classification harder. This selection strategy resulted in five potential biomarkers for IAPV infection (Figure 2A) and three potential biomarkers for SBPV infection (Figure 2B). Together, these eight components were further assigned as the “untargeted biomarker set”. 

### 3.2. Targeted Data Analysis

**Figure 3:**
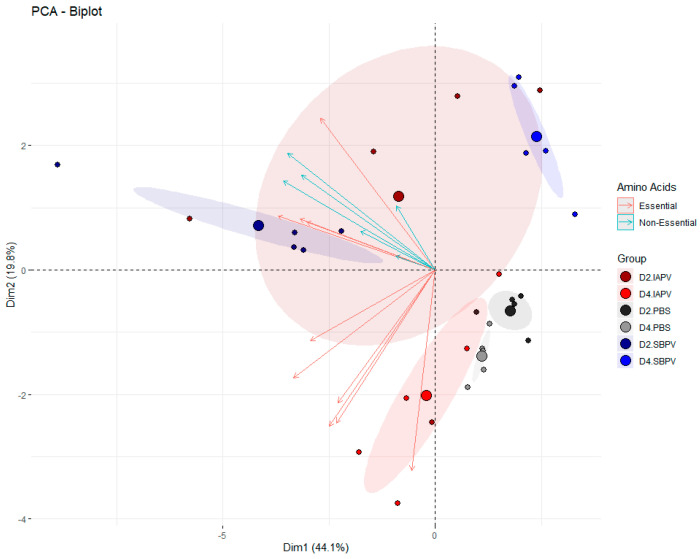
Principal component analysis (PCA-X) of essential and non-essential amino acids in relation to virus infection (SBPV or IAPV) and exposure time (2DPI and 4DPI) in bees with a normal food regime (50% sugar). Small dots represent the actual datapoints; the bigger dots represent the center of the ellipses with a 95% confidence level.

Based on our in-house library of 300 metabolites, we were able to identify 76 metabolites in the hemolymph of commercial bees (for an overview of these metabolites, see supplementary information Table S1). The identity of the metabolites allowed us to make an informed decision on the potential of a discriminating metabolite as a biomarker, by providing a biological insight on what is happening in the infected bee, for instance, whether there is a direct or indirect mechanistic link to the effects of the virus or if the marker is also associated with other stressors of bees. We followed a three-step approach to select biologically relevant biomarkers: (1) metabolites were selected that were significantly correlated to infection stage, (2) on this list of metabolites a pathway analysis was performed, and (3) a literature search was performed to identify potential viral specific signatures. The results are summarized in supplementary information S1 (Tables S2 and S3 and Figure S1). The majority of the virus-disturbed metabolic pathways are related to amino acid biosynthesis, amino acid degradation, and aminoacyl-tRNA biosynthesis (supplementary information S1, Tables S2 and S3). Amino acids are key components in bee hemolymph [35,36], and in total we identified 17 amino acids in the hemolymph. Principal component analysis with these 17 amino acids showed that viral infection stadia can be separated, and essential and non-essential amino acids vectors are correlated within but not amongst each (Figure 3). The ratio of essential and non-essential amino acids is thus disturbed in relation to virus infection, with amino acid metabolism known to be important for viral replication [23,37]. It has been reported that viruses can modify the host amino acids production to promote viral genome replication [22]. Such a stimulus could result in altered ratios of essential versus non-essential amino acids (the latter being those produced by the host). 

A second important group of metabolites we observed were the polyamines: spermidine, spermine, cadaverine, and putrescine, to see if they were differentially expressed (supplementary information S1 Figure S1). They were included as biological relevant markers, as dysregulation of cellular polyamines have been associated with various pathological conditions including viral infections [38]. Polyamines play a role in viral entry, transcription, replication, and virion packaging [39]. They neutralize negative charges, which are useful to package the negatively charged viral genome into the virion [40]. From the host’s perspective, polyamines play a role in nucleotide and lipid metabolism and reactive oxygen species production [39]. We see no clear pattern in levels of polyamines in relation to virus and infection stage; this can be explained as we cannot differentiate between the polyamines sequestered in the virions and those available to host.

Together, these 21 components (17 amino acids and 4 polyamines) are further on named as the “targeted biomarker set”. In the commercial bees, the AUC of this biomarker set to identify a viral infection was 0.876 (95% CI: 0.783–0.949).

### 3.3. Predictive Power of the Biomarker Set

In Table 2, we describe the predictive power, with the classifying algorithm random forest, in a group of 40 wild bees. The untargeted and targeted biomarker set had good diagnostic performance for discriminating IAPV, SBPV, and general virus infection in standardized commercial bees. Both sensitivity (ratio of true positives over the sum of true and false positives) and precision (ratio of true positives over the sum of true positives and false negatives) increased when we used the targeted biomarkers, yet this effect could be due to the higher number of components in this set. Overall, the marker signatures had good classification power, identifying viral infection but also being able to differentiate between a virulent IAPV and a less virulent SBPV infection. We would like to point-out that classification was not based on absolute concentrations or thresholds of the suggested markers, and that wild and standardized bees had different levels of the biomarkers. 

## 4. Conclusions

In summary, this study provided the first evidence of biomarker discovery for predicting virus infection in bees. A dual targeted and untargeted metabolomics approach was established to analyze both covert and overt virus infection in *B. terrestris*. Twenty-nine metabolites (i.e., 21 identified and eight unidentified metabolites) were finally selected as potential biomarkers, descriptive for virus infection. There is growing evidence that the impacts of viruses on their bee hosts can be exacerbated by poor nutrition [33,41]; hence, a nutritional stress parameter was incorporated. It remains to be determined if the biomarker sets can differentiate natural infections in different environments encompassing different stressors impacting general bee health. Yet, we were able to demonstrate good diagnostic performance in standardized and wild bees in which these stressors were artificially induced. Our results showed the potential of a metabolic biomarker-based approach to classify virus infection. Moving forward, large-scale independent validation studies conducted in laboratory and field settings using independent cohorts are necessary to refine and validate our metabolomics-based biomarkers of virus infection in bees. Datasets linking information of the bee hemolymph metabolome and its physiological status, together with virus titers and virus tissue tropisms, will further improve our understanding of the impact of different viruses in different bee species. The next step is to describe these complex multi-host, multi-virus assemblies. Unlike transcriptome or proteome markers, which are diversified from species to species, metabolite composition is highly similar between species. This underlines the potential of metabolite biomarkers to be adequate in a broader set of bees, and to function as tools to describe the health of a community foraging within a specific environment. 

## Figures and Tables

**Table 1:** Classification dataset composition and specification of constructed Orthogonal Projections to Latent Structures Discriminant Analysis (OPLS-DA) models, with output of the model validation procedure. OPLS-DA models were based on quality control (QC)-normalized data.

Model	Comparison	Model Specification	Numbers of Model Components (t_o_ + t_p_) ^a^	Model Characteristics ^b^	*p*-value Cross-Validated ANOVA ^c^	Permutation ^d^
	*** Virus ***					
* 1 *	25% diet: virus (*n* = 25) vs. non-virus (*n* = 30)	Virus	- ^e^	-	-	No
* 2 *	50% diet: virus (*n* = 25) vs. non-virus (*n* = 30)	Virus	1 + 4 + 0	R^2^Y = 0.97; Q^2^ = 0.741	6.10 × 10^−10^	Good
* 3 *	All: virus (*n* = 50) vs. non-virus (*n* = 60)	Virus	-	-	-	No
	*** IAPV ***					
* 4 *	25% diet: IAPV (*n* = 10) vs. PBS (*n* = 10)	IAPV	-	-	-	No
* 5 *	50% diet: IAPV (*n* = 10) vs. PBS (*n* = 10)	IAPV	1 + 3 + 0	R^2^Y = 0.995; Q^2^ = 0.906	2.80 × 10^−5^	Good
* 6 *	All: 4DPI (*n* = 10) vs. 2DPI (*n* = 10)	Time	1 + 5 + 0	R^2^Y = 0.999; Q^2^ = 0.875	1.75 × 10^−3^	Good
* 7 *	All: IAPV (*n* = 20) vs. PBS (*n* = 20)	IAPV	1 + 5 + 0	R^2^Y = 0.993; Q^2^ = 0.905	1.01 × 10^−10^	Good
	*** SBPV ***					
* 8 *	25% diet: SBPV (*n* = 15) vs. PBS (*n* = 15)	SBPV	-	-	-	No
* 9 *	50% diet: SBPV (*n* = 15) vs. PBS (*n* = 15)	SBPV	1 + 4 + 0	R^2^Y = 0.995; Q^2^ = 0.931	6.49 × 10^−9^	Good
* 10 *	All: 4DPI (*n* = 10) vs. 2DPI (*n* = 10)	Time	1 + 4 + 0	R^2^Y = 0.997; Q^2^ = 0.883	3.81 × 10^−4^	Good
* 11 *	All: 14DPI (*n* = 10) vs. 2DPI (*n* = 10)	Time	1 + 2 + 0	R^2^Y = 0.970; Q^2^ = 0.871	4.11 × 10^−5^	Good
* 12 *	All: 14DPI (*n* = 10) vs. 4DPI (*n* = 10)	Time	1 + 7 + 0	R^2^Y = 1.000; Q^2^ = 0.863	2.00 × 10^−2^	Good
* 13 *	All: SBPV (*n* = 30) vs. PBS (*n* = 30)	SBPV	1 + 9 + 0	R^2^Y = 0.996; Q^2^ = 0.694	3.66×10^−5^	Good
	*** IAPV vs. SBPV ***					
* 14 *	25% diet: SBPV (*n* = 10) vs. IAPV (*n* = 10)	SBPV/IAPV	1 + 4 + 0	R^2^Y = 0.996; Q^2^ = 0.827	2.77 × 10^−3^	Good
* 15 *	50% diet: SBPV (*n* = 10) vs. IAPV (*n* = 10)	SBPV/IAPV	1 + 1 + 0	R^2^Y = 0.958; Q^2^ = 0.849	5.14 × 10^−6^	Good
* 16 *	All: SBPV (*n* = 30) vs. IAPV (*n* = 20)	SBPV/IAPV	1 + 5 + 0	R^2^Y = 0.984; Q^2^ = 0.811	5.67 × 10^−10^	Good

^a^ with the orthogonal and the predictive component; ^b^ with R^2^Y the variation in Y that is explained by the model, and Q^2^ the predictive ability of the model. Q^2^ > 0.5 indicated good model quality [32]; ^c^ a cross-validated ANOVA *p*-value < 0.05 indicated good model quality; ^d^ good permutation testing was achieved if R^2^Y and Q^2^ values of the models based on the permutated data were significantly lower than those based on the real data set; ^e^ this model cannot be validated.

**Table 2:** Performance of diagnostic support models with Random Forest constructed using different candidate biomarkers for wild bees.

Features	Discrimination	Sensitivity	Precision	AUC
*Untargeted (n = 8)*	Virus vs. Non-virus	77.5%	77.6%	0.892
	IAPV vs. Non-virus	86.7%	88.9%	0.955
	SBPV vs. Non-virus	80.0%	79.5	0.815
*Amino acids + polyamines (n = 21)*	Virus vs. Non-virus	90.0%	90.0%	0.945
	IAPV vs. Non-virus	93.3%	93.3%	0.985
	SBPV vs. Non-virus	90.0%	89.9%	0.875
*Combined (n = 29)*	Virus vs. Non-virus	90.0%	90.0%	0.948
	IAPV vs. Non-virus	90.0%	89.9%	0.985
	SBPV vs. Non-virus	83.3%	83.1%	0.930

The normalized mass spectral ion intensities of the untargeted and targeted biomarker candidates were used, marking IAPV, SBPV and non-virus infection. Ten-fold cross-validation was used.

## Supplementary Materials

The following are available online at https://www.mdpi.com/article/10.3390/v13040600/s1, Figure S1: Abundance analysis of four polyamines detected in 50% sugar syrup-fed bee hemolymph. Table S1: Peak abundance ratio of identified metabolites in bee hemolymph between 25% sugar syrup and control, Table S2: Significantly disturbed pathways in IAPV-infected bees (IIB), Table S3: Significantly disturbed pathways in SBPV-infected bees (SIB).
