# Supplementary material for: The Bee Hemolymph Metabolome: A Window into the Impact of Viruses on Bumble Bees
Source: Viruses. 2021 Apr 1;13(4):600. doi: 10.3390/v13040600 (PMC8066158; doi:10.3390/v13040600)
Supplement: Supplementary file 1 [file viruses-13-00600-s001.pdf]

viruses

MDPI

Article

The Bee Hemolymph Metabolome: A Window into the Impact of Viruses on Bumble Bees

Luoluo Wang [1,2], Lieven Van Meulebroek [3], Lynn Vanhaecke [3], Guy Smagghe [2] and Ivan Meeus [2,*]

1 Guangdong Provincial Key Laboratory of Insect Developmental Biology and Applied Technology, Institute of Insect Science and Technology, School of Life Sciences, South China Normal University, Guangzhou, China; Luoluo.Wang@scnu.edu.cn
2 Department of Plants and Crops, Faculty of Bioscience Engineering, Ghent University, Ghent, Belgium; Guy.Smagghe@UGent.be, Ivan.Meeus@UGent.be
3 Laboratory of Chemical Analysis, Department of Veterinary Public Health and Food Safety, Faculty of Veterinary Medicine, Ghent University, Merelbeke, Belgium; Lieven.Vanmeulenbroek@UGent.be; Lynn.Vanhaecke@UGent.be
* Correspondence: Ivan.Meeus@UGent.be

Citation: Wang, L.L.; Van Meulebroek, L.; Vanhaecke. L.; Smagghe, G.; Meeus, I.; The Bee Hemolymph Metabolome: A Window into the Impact of Viruses on Bumble Bees. *Viruses* **2021**, *13*, 600. https://doi.org/10.3390/v13040600

Academic Editor: Michelle Flenniken

Received: 04 March 2021
Accepted: 30 March 2021
Published: 1 April 2021

Publisher's Note: MDPI stays neutral with regard to jurisdictional claims in published maps and institutional affiliations.

Copyright: © 2021 by the authors. Submitted for possible open access publication under the terms and conditions of the Creative Commons Attribution (CC BY) license (http://creativecommons.org/licenses/by/4.0/).

Selection of the targeted biomarker set:

In total we identified 76 metabolites, including 28 amino acids (37%), 11 carbohydrates (14%), 11 carboxylic acids, 2 TCA intermediates, 4 polyamines, 4 nucleic acids, and 16 compounds from other chemical classes (Table S1). We selected biologically-relevant biomarker candidates based on a three step approach: (1) their expression profile in standardized bees and its relation with viral presence, (2) pathways analysis on significant metabolites; and (3) a literature search to identify potential viral specific signatures.

Step (1) and (2), pathways analysis on significant metabolites

We performed two-way ANOVA with Tukey HSD tests for post-hoc comparisons and used significant metabolites for metabolic pathway analysis using the web-based platform MetaboAnalyst (http://www.metaboanalyst.ca/) in order to get insights in the biological relevance of the observed changes. The made comparisons and significant metabolites are summarized in Table S2 and S3, showing three pathways including D-Glutamine and D-Glutamate metabolism, Arginine and proline metabolism and Aminoacyl-tRNA biosynthesis that were significantly modulated in both virus-infected bees. These disturbed pathways were mainly linked with the early infection stages of SBPV infection (2DPI and 4DPI), and multiple stages of IAPV infection. As a matter of fact, the majority of disturbed pathways was related to amino acid biosynthesis, amino acid degradation, and aminoacyl-tRNA biosynthesis.

Step [3], mechanistic qualification of potential biomarkers in relation to literature

Amino acid metabolism is known to have an important role in viral replication, and amino acids can mediate the metabolic crosstalk between host and virus [1]. For instance, virus can increase the host amino acids production for rapid viral genome replication via modifying the host metabolism [2]. Moreover, amino acids are very abundant components of the bee hemolymph [3, 4], and thus suitable markers for monitoring. Hence, a special interest went to amino acids, in particular to the different responses of essential and non-essential amino acids in the bee hemolymph upon virus infection (see Figure 3, main manuscript).

We observed 4 polyamines, spermidine, spermine, cadaverine, and putrescine in bee hemolymph, for which differential expression related to viral infection was observed (Figure S1). Polyamines are important for nucleotide charge neutralization [5]. Dysregulation of cellular polyamines was associated with various pathological conditions including viral infections [5]. Moreover, several studies indicated a significant role for polyamines in the replication of RNA viruses [6-8].

Table S1 Peak abundance ratio of identified metabolites in bee hemolymph between 25% sugar syrup and control. Ratios were based on QC-normalized data.

Metabolites	Mock 25%/50% ratio	PBS 25%/50% ratio	SBPV 25%/50% ratio	IAPV 25%/50% ratio
Carbohydrates				
Sucrose	1.07	1.16	1.65	0.89
D-Glucose	0.53	1.36	1.36	0.67
Alpha-Lactose	1.20	1.21	1.36	0.79
D-Maltose	1.07	1.16	1.65	0.89
D-Ribose	0.93	1.24	0.56	1.45
D-Arabinose	0.93	1.24	0.56	1.45
D-Pinitol	0.62	0.57	0.41	4.07
Glucitol	1.15	0.96	0.75	0.78
Glycerol	1.11	1.23	0.80	0.84
Mannitol	1.15	0.96	0.75	0.78
Glyceraldehyde	0.59	1.74	3.90	0.69
Amino acids				
L-Ornithine	1.14	0.80	0.92	1.42
L-Lysine (Lys)	0.89	0.85	0.67	1.25
L-Arginine (Arg)	0.91	1.10	0.58	1.20
L-Histidine (His)	1.08	0.98	0.91	1.11
L-Asparagine (Asn)	0.85	0.94	0.51	1.09
L-Serine (Ser)	0.68	0.83	0.35	0.94
L-Aspartic acid (Asp)	0.86	1.24	0.94	0.91
L-Glutamine (Gln)	0.74	0.80	0.41	1.53
L-Glutamic acid (Glu)	1.06	1.25	1.33	0.96
4-Hydroxyproline	0.89	0.85	0.60	1.33
D-Alanine (Ala)	0.82	0.78	0.76	0.70
2-Aminoisobutyric acid	0.66	0.94	0.41	1.72
L-Proline (Pro)	1.00	0.92	1.08	1.12
L-Threonine (Thr)	0.70	0.97	0.62	1.08
L-Valine (Val)	0.86	0.86	0.79	1.03
Acetylcarnitine	1.01	0.87	0.75	0.89
Methionine (Met)	0.88	1.14	0.75	1.25
Diaminopimelic acid	1.12	0.83	0.70	1.18
Gamma-Aminobutyric acid	0.66	0.94	0.41	1.72
5-Aminovaleric acid	0.86	0.86	0.79	1.03
Pyroglutamic acid	0.99	0.65	0.36	1.79
N-Acetylglutamic acid	1.01	0.65	0.71	1.72
L-Tyrosine (Tyr)	0.93	0.88	0.83	1.06
L-Isoleucine (IIe)	0.99	0.96	0.86	0.89
L-Leucine (Leu)	0.99	0.96	0.86	0.89
L-Phenylalanine (Phe)	0.91	0.75	0.80	1.04
N-acetyl-L-methionine	0.88	1.03	0.54	1.13
Tyramine	1.59	0.59	1.23	1.18
Polyamines				
Spermidine	1.03	0.87	0.95	1.18
Spermine	1.06	0.85	0.86	1.11
Putrescine	0.94	0.95	1.05	1.06
Cadaverine	0.83	0.43	0.58	1.31

Nucleotides				
Uracil	1.14	1.02	1.10	0.95
Inosine	0.91	0.85	0.85	0.99
Cytosine	1.06	0.95	1.46	4.17
Hypoxanthine	0.84	0.99	0.62	6.62
TCA intermediates				
Alpha-Ketoglutaric acid	1.13	0.82	1.18	0.97
Citric acid	1.56	1.31	1.11	2.84
Carboxylic acid				
Gluconic acid	1.02	1.64	1.19	15.97
Thiazolidine-2-carboxylic acid	1.09	1.16	0.59	1.58
Ascorbic acid	1.53	0.79	1.15	1.18
Saccharic acid	1.06	0.71	0.58	1.42
Pipecolic acid	0.87	0.77	0.31	2.44
Sebacic acid	0.74	0.98	1.00	3.97
Hydrocinnamic acid	0.97	0.97	0.99	1.10
4-Hydroxyphenylacetic acid	0.24	3.03	1.08	0.99
Vanillic acid	0.33	1.94	0.98	1.01
Other chemical classes				
Linolenic acid	1.49	0.66	2.68	0.76
2,3-Pentanedione	0.91	0.88	0.73	1.59
Imidazolepropionic acid	0.58	1.15	0.35	1.76
Urocanic acid	0.90	0.64	0.87	0.65
Pantothenic acid	0.40	0.48	0.53	1.77
3-Methyl-2-cyclohexen-1-one	0.98	1.23	1.02	1.20
Nicotinic acid	1.02	0.93	1.08	1.37
3-Hydroxybutyric acid	0.80	0.53	0.53	1.81
4-Methylcatechol	0.87	1.08	0.96	1.79
Azaleic acid	0.84	1.23	0.94	1.37
Ethanolamine	4.99	0.66	1.06	0.86
Glycerol-1-phosphate	0.95	1.57	1.39	0.86
Phosphoenolpyruvic acid	1.92	0.93	1.09	1.08
Cyclohexylamine	0.98	0.72	1.09	1.24
Tryptamine	0.80	1.09	1.12	0.88
Sarcosine	0.83	0.82	0.74	0.67
3,4-Dihydroxyphenylacetic acid	0.33	1.88	0.96	1.02
2-Hydroxyphenylacetic acid	0.24	3.32	1.00	1.02

Table S2. Significantly disturbed pathways in IAPV-infected bees (IIB)

Samples	Pathway name	Hits	*P*-value	Impact	Significant Metabolites linked with a specific pathway
**IIB-50-2DPI**	D-Glutamine and D-glutamate metabolism	2/5	3.58E-03	1.00	L-Glutamic acid, Alpha-Ketoglutaric acid;
	Arginine and proline metabolism	4/37	4.81E-03	0.44	L-Arginine, L-Ornithine, 4-Hydroxyproline, L-Glutamic acid;
	Aminoacyl-tRNA biosynthesis	4/67	3.79E-02	0.14	L-Arginine, L-Glutamic acid, L-Serine, L-Histidine;

**IIB-50-4DPI**	Alanine, aspartate and glutamate metabolism	3/23	6.69E-03	0.19	L-Glutamic acid, Gamma-Aminobutyric acid, L-Asparagine;
	Aminoacyl-tRNA biosynthesis	4/67	2.64E-02	0.14	L-Arginine, L-Asparagine, L-Serine, L-Histidine;
	Starch and sucrose metabolism	2/17	3.46E-02	0.07	Sucrose, D-Maltose;
	Citrate cycle (TCA cycle)	2/20	4.69E-02	0.07	Phosphoenolpyruvic acid, alpha-Ketoglutaric acid;
**IIB-25-2DPI**	Aminoacyl-tRNA biosynthesis	8/67	5.35E-05	0.14	L-Asparagine, L-Histidine, L-Arginine, L-Glutamine, L-Serine, L-Methionine, L-Lysine, L-Glutamic acid;
	Arginine and proline metabolism	7/37	1.01E-04	0.43	L-Glutamine, L-Arginine, L-Ornithine, L-Glutamic acid, Spermidine, Gamma-Aminobutyric acid;
	Glutathione metabolism	5/26	1.83E-04	0.13	L-Glutamic acid, Pyroglutamic acid, Ornithine, Spermidine, Cadaverine;
	Alanine, aspartate and glutamate metabolism	4/23	1.35E-03	0.58	L-Glutamine, L-Glutamic acid, Gamma-Aminobutyric acid, L-Asparagine;
	D-Glutamine and D-glutamate metabolism	2/5	4.73E-03	1.00	L-Glutamine, Alpha-Ketoglutaric acid;
	Nitrogen metabolism	2/7	9.67E-03	0.00	L-Glutamine, Alpha-Ketoglutaric acid;
	Galactose metabolism	3/26	1.89E-02	0.09	Alpha-Lactose, D-Glucose, Glycerol;
**IIB-25-4DPI**	Pantothenate and CoA biosynthesis	3/12	3.17E-03	0.00	Pantothenic acid, L-Valine, Uracil;
	Valine, leucine and isoleucine biosynthesis	3/14	4.05E-03	1.00	L-Leucine, L-Valine, L-Isoleucine;
	Aminoacyl-tRNA biosynthesis	6/67	6.55E-03	0.14	L-Histidine, L-Serine, L-Valine, L-Lysine, L-Isoleucine, L-Leucine;

Pairwise metabolite pathway analysis was conducted on all significant metabolites linked with a specific comparison. Pathways with highest pathway impact values were considered the most significantly affected pathways.

Table S3. Significantly disturbed pathways in SBPV-infected bees (SIB)

**Samples**	**Pathway name**	**Hits**	***P*-value**	**Impact**	**Significant Metabolites linked with a specific pathway**
**SIB-50-2DPI**	Aminoacyl-tRNA biosynthesis	13/67	3.04E-07	0.14	L-Arginine, L-Asparagine, L-Histidine, L-Serine, L-Isoleucine, L-Leucine, L-Lysine, L-Phenylalanine, L-Proline, L-Threonine, L-Tyrosine, L-Valine, L-Glutamine
	Arginine and proline metabolism	7/37	3.18E-04	0.30	L-Glutamine, L-Arginine, L-Proline, Spermidine, Hydroxyproline, Gamma-Aminobutyric acid, Spermine;
	Valine, leucine and isoleucine biosynthesis	4/14	1.05E-03	1.00	L-Threonine, L-Leucine, L-Isoleucine, L-Valine;
**SIB-50-4DPI**	Phenylalanine, tyrosine and tryptophan biosynthesis	2/4	8.26E-03	1.00	L-Phenylalanine, L-Tyrosine;
	Alanine, aspartate and glutamate metabolism	4/23	9.80E-03	0.37	alpha-Ketoglutaric acid, Gamma-Aminobutyric acid, L-Asparagine, L-Glutamine;
	D-Glutamine and D-glutamate metabolism	2/5	1.34E-02	0.00	L-Glutamine, alpha-Ketoglutaric acid;
	Aminoacyl-tRNA biosynthesis	10/67	1.41E-04	0.14	L-Phenylalanine, L-Arginine, L-Serine, L-Methionine, L-Valine, L-Isoleucine, L-Leucine, L-Threonine, L-Tyrosine, L-Proline;
	Valine, leucine and isoleucine biosynthesis	4/13	1.15E-03	1.00	L-Threonine, L-Leucine, L-Isoleucine, L-Valine;
	Arginine and proline metabolism	6/37	2.42E-03	0.30	L-Arginine, L-Proline, Spermidine, Spermine, Putrescine, Gamma-Aminobutyric acid;
	Glutathione metabolism	5/26	2.66E-03	0.03	Pyroglutamic acid, Putrescine, Spermidine, Cadaverine, Spermine;

**SIB-50-14DPI**	Phenylalanine, tyrosine and tryptophan biosynthesis	2/4	8.68E-03	1.00	L-Phenylalanine, L-Tyrosine;
	Aminoacyl-tRNA biosynthesis	7/67	2.25E-04	0.00	L-Histidine, L-Arginine, L-Aspartic acid, L-Methionine, L-Valine, L-Lysine, L-Threonine;
	Starch and sucrose metabolism	3/17	4.34E-03	0.10	Sucrose, D-Maltose, D-Glucose;
	Pantothenate and CoA biosynthesis	2/12	2.38E-02	0.00	Pantothenic acid, L-Arginine;
**SIB-25-2DPI**	Alanine, aspartate and glutamate metabolism	2/23	4.73E-03	0.22	L-Aspartic acid, alpha-Ketoglutaric acid;
	Arginine and proline metabolism	2/37	1.21E-02	0.16	L-Aspartic acid, L-Ornithine;
	D-Glutamine and D-glutamate metabolism	1/5	2.45E-02	0.00	alpha-Ketoglutaric acid;
**SIB-25-4DPI**	Aminoacyl-tRNA biosynthesis	3/67	3.96E-0	0.00	L-Histidine, L-Arginine, L-Lysine;
**SIB-25-14DPI**	Aminoacyl-tRNA biosynthesis	3/67	1.74E-02	0.00	L-Histidine, L-Methionine, L-Lysine;
	Biotin metabolism	1/5	4.37E-02	0.00	L-Lysine;

Pairwise metabolite pathway analysis was conducted on all significant metabolites linked with a specific comparison. Pathways with highest pathway impact values were considered the most significantly affected pathways.

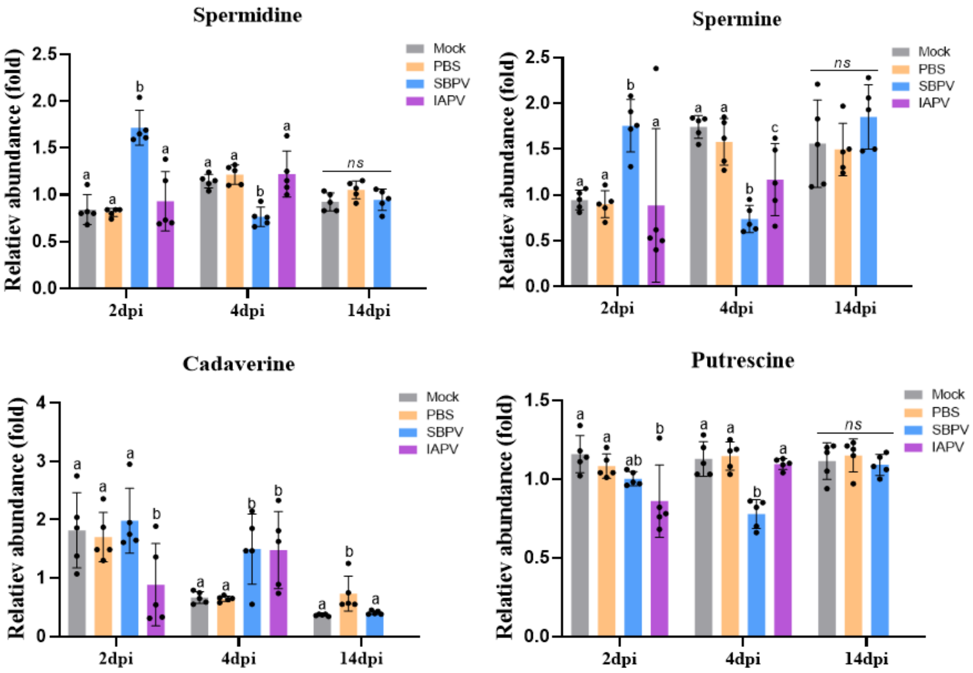

Figure S1: Abundance analysis of four polyamines detected in 50% sugar syrup-fed bee hemolymph. The data shown are mean±SE (n=5). Different letters indicate significant differences within the same time point ($P < 0.05$, One-way ANOVA followed with Tukey's HST test). Two key polyamines, spermidine and spermine were significantly increased (P-value < 0.05) at the early stages of SBPV infection, followed by a significant decrease at the early-middle stages, and finally recovered to the normal levels as compared to PBS controls. Cadaverine was significantly increased (P-value < 0.05) at early-middle stages followed by backing to normal levels at late stages of SBPV infected bees. Putrescine was also significantly decreased (P-value < 0.05) at the early-middle stages of SBPV infection. However, in IAPV infected bees, only cadaverine was altered, with a significant increase (P-value < 0.05) at the late stage.

References

1. Ren, W.; Rajendran, R.; Zhao, Y.; Tan, B.; Wu, G.; Bazer, F.W.; Zhu, G.; Peng, Y.; Huang, X.; Deng, J. Amino acids as mediators of metabolic cross talk between host and pathogen. *Front. Immunol.* **2018,** 9: 319.
2. Mayer, K.A.; Stockl, J.; Zlabinger, G.J.; Gualdoni, G.A. Hijacking the Supplies: Metabolism as a Novel Facet of Virus-Host Interaction. *Front. Immunol.* **2019**, 10: 1533.
3. Wyatt, G.R. The biochemistry of insect hemolymph. *Annu. Rev. Entomol.* **1961**, 6: 75-102.
4. Wyatt, G.R.; Kalf, G.F. The chemistry of insect hemolymph: II. Trehalose and other carbohydrates. *J. Gene. Physiol.* **1957**, 40: 833-847.
5. Mounce, B.C.; Olsen, M.E.; Vignuzzi, M.; Connor, J.H. Polyamines and their role in virus infection. *Microbiol Mol. Biol. Rev.* **2017**, 81: e00029-17.
6. Li, M.H.; MacDonald, M.R. Polyamines: small molecules with a big role in promoting virus Infection. *Cell Host Microbe* **2016**, 20: 123-134.
7. Tuomi, K.; Raina, A.; Mäntyjärvi, R. Synthesis of *Semliki forest virus* in polyamine depleted baby hamster kidney cells. *Biochem. J* **1982**, 206: 113-119.
8. Raina, A.; Tuomi, K.; Mäntyjärvi, R. Roles of polyamines in the replication of animal viruses. *Med. Biol.* 1981, 59: 428-432.
